# Molecular and cellular mechanisms controlling integrin-mediated cell adhesion and tumor progression in ovarian cancer metastasis: a review

**DOI:** 10.1007/s10585-021-10136-5

**Published:** 2021-11-25

**Authors:** Dolly Dhaliwal, Trevor G. Shepherd

**Affiliations:** 1 The Mary & John Knight Translational Ovarian Cancer Research Unit, Lawson Health Research Institute and London Health Sciences Centre, London, ON Canada; 2 Department of Anatomy & Cell Biology, Schulich School of Medicine & Dentistry, Western University, London, ON Canada; 3 Department of Obstetrics & Gynaecology, Schulich School of Medicine & Dentistry, Western University, London, ON Canada; 4 Department of Oncology, Schulich School of Medicine & Dentistry, Western University, London, ON Canada; 5 London Regional Cancer Program, 790 Commissioners Rd E, Room A4-836, London, ON N6A 4L6 Canada

**Keywords:** Ovarian cancer, Integrins, Metastasis, Cell adhesion, Tumor progression

## Abstract

Epithelial ovarian cancer (EOC) is the most lethal gynecological malignancy in the developed world. EOC metastasis is unique since malignant cells detach directly from the primary tumor site into the abdominal fluid and form multicellular aggregates, called spheroids, that possess enhanced survival mechanisms while in suspension. As such, altered cell adhesion properties are paramount to EOC metastasis with cell detachment from the primary tumor, dissemination as spheroids, and reattachment to peritoneal surfaces for secondary tumor formation. The ability for EOC cells to establish and maintain cell–cell contacts in spheroids is critical for cell survival in suspension. Integrins are a family of cell adhesion receptors that play a crucial role in cell–cell and cell-extracellular matrix interactions. These glycoprotein receptors regulate diverse functions in tumor cells and are implicated in multiple steps of cancer progression. Altered integrin expression is detected in numerous carcinomas, where they play a role in cell migration, invasion, and anchorage-independent survival. Like that observed for other carcinomas, epithelial-mesenchymal transition (EMT) occurs during metastasis and integrins can function in this process as well. Herein, we provide a review of the evidence for integrin-mediated cell adhesion mechanisms impacting steps of EOC metastasis. Taken together, targeting integrin function may represent a potential therapeutic strategy to inhibit progression of advanced EOC.

## Introduction

### Epithelial ovarian cancer

Epithelial ovarian cancer (EOC) is the most lethal gynecological malignancy in the developed world [1]. Most women are diagnosed with advanced-stage disease with a 5-year survival rate of less than 29%, since 80% of these cases present initially with metastasis [1, 2]. The delay in diagnosis can be attributed to the wide range of non-specific symptoms like abdominal fullness, vaginal bleeding and urinary symptoms thereby leading to a more advanced-stage before clinical assessment [3]. Factors for increased risk include early age of menarche, and benign gynecological conditions such as endometriosis, polycystic ovary and pelvic inflammatory disease, whereas oral contraceptive use and tubal ligation can decrease risk for EOC [1]. The current treatment plan for patients with EOC in which tumors have spread beyond the ovaries is maximal surgical cytoreduction with adjuvant chemotherapy of combined carboplatin and paclitaxel [1]. However, 75% of patients will have disease reoccurrence, oftentimes acquiring chemotherapy resistance, ultimately leading to a dire prognosis [1, 3].

EOC encompasses a heterogenous group of malignant tumors that differ in prognosis, molecular pathology, and etiology. According to the World Health Organization (WHO), EOC can be classified into seven histological subtypes: high-grade and low-grade serous, mucinous, endometrioid, clear cell, Brenner, seromucinous and undifferentiated carcinomas [4]. These histological subtypes can be organized into two major EOC groups where Type I consists of lower grade, slow-proliferating carcinomas within serous, endometrioid, mucinous and clear cell histological subtypes that likely arise from benign ovarian lesions [4, 5]. Whereas Type II tumors are typified as being more aggressive disease derived from secretory fallopian tube epithelium, and present histologically as high-grade serous, mixed epithelial or undifferentiated carcinomas [5, 6]. High-grade serous ovarian cancer (HGSOC) represents 75% of all cases and is characterized by the near universal presence of *TP53* tumor suppressor gene mutations, commonly as observed as missense gain-of-function alterations, although deletions and nonsense loss-of-function mutations have been identified, too [7, 8]. This genetic alteration arises within an early tumor precursor cell of the distal fallopian tube, called serous tubal intraepithelial carcinoma (STIC) lesion; protein-stabilizing *TP53* missense mutations promote secretory epithelial cell survival and cell–cell aggregation under anchorage independent growth conditions [8]. HGSOC is associated with lower prevalent but recurrent somatic mutations in *NF1*, *BRCA1*, *BRCA2*, *RB1* and *CDK12* totalling 5–8% of tumors [1, 8].

Furthermore, advanced EOC is characterized by ascites fluid accumulation within the peritoneal cavity [5]. The impairment of lymphatic drainage and increased secretion of vascular endothelial growth factor (VEGF) leads to enhanced vascular permeability [5]. The unique microenvironment within malignant EOC ascites consists of a variety of non-tumor cell types, such as fibroblasts, mesothelial cells, immune cells and endothelial cells, as well as acellular components, such as soluble extracellular matrix (ECM), matrix-degrading enzymes, cytokines and growth factors [9].

The ECM is an integral and dynamic non-cellular component within all tissues and functions to support cells and maintain tissue homeostasis [9–11]. Normal ovarian ECM is composed of a highly-ordered arrangement of collagen fibers and proteoglycans, such as decorin and versican, to provide structural integrity, and maintain interstitial pressure and hydration to tissue [9–11]. However, ECM stiffness is commonly increased in EOC tumors through the activation of stromal fibroblasts and collagen remodeling into thick fibrils in random orientation, which can combine to increase tumorigenesis, cancer invasion and migration [10–12]. For example, decorin loss and upregulation of versican, fibronectin, tenascin-C, and tenascin-X are associated with poor prognosis and overall survival in EOC [9, 12]. The binding of various ECM ligands to integrins, which are glycoprotein receptors at the cell surface to promote adhesion, regulate complex signaling events alone or in combination with growth factor receptors [13, 14].

The interactions between the tumor cells and ECM within the tumor microenvironment are crucial since their dysregulation has been implicated in EOC progression [9, 13]. Therefore, integrin-mediated interactions and function within the tumor micro-environment represents a potential unique therapeutic strategy in EOC. In this review, we discuss the contributions of integrin-mediated cell adhesion in the critical steps during intraperitoneal metastatic cascade of EOC pathogenesis, including spheroid formation, epithelial-mesenchymal plasticity, and mesothelial attachment of secondary tumors.

### Integrin signalling

**Fig. 1:**
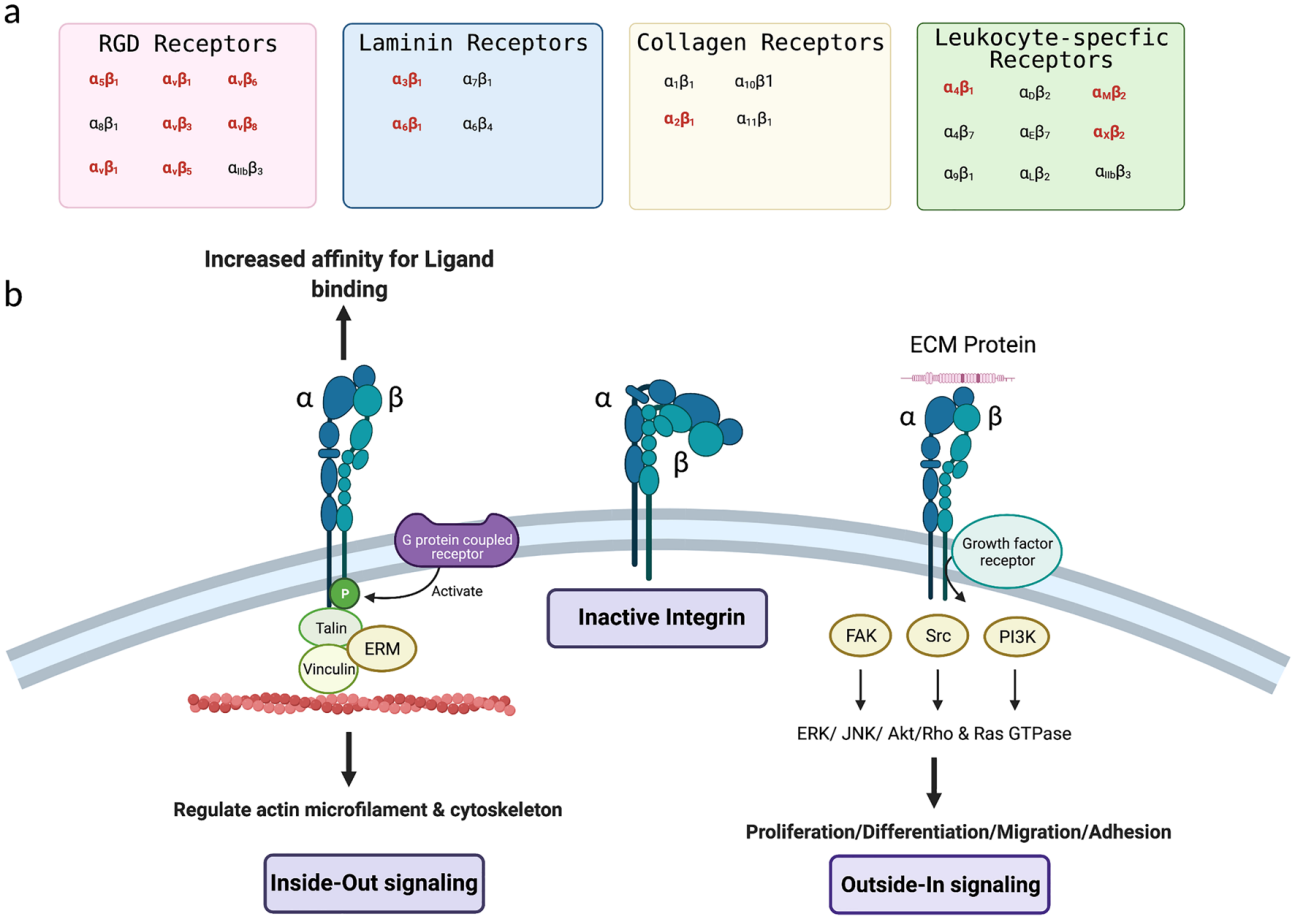
Integrins are a superfamily of cell adhesion receptors that recognize ECM and cell-surface ligands. They consist of 18 $\mathrm{\alpha }$-subunits and 8 β-subunits that assemble to create 24 functionally distinct transmembrane heterodimers. **a** Integrins are grouped according to their ligand-binding specificity: arginine-glycine-aspartate (RGD)-recognizing integrins, laminin-binding integrins, collagen-binding integrins and leukocyte-integrins. **b** Integrins take part in bidirectional signaling once the integrins move from a bent configuration to an active form. During inside-out signaling, G-protein coupled receptors lead to integrin β subunit phosphorylation within the cytoplasmic domain for receptor activation. The integrin binding of cytoskeletal proteins such as talin, vinculin and ERM (ezrin, radixin, and moesin) acts to regulate actin microfilaments of the cytoskeleton. Outside-in signaling occurs through the clustering of integrin receptors at the plasma membrane where ECM ligation transduces signals intracellularly. ECM binding with ligands such as collagen, laminin and fibronectin induces conformational changes in the integrin receptor to allow intracellular tails of the β subunit to engage with intracellular signaling molecules including focal adhesion kinase (FAK), small GTPases Rho and Ras, and adaptors, such as Cas/Crk and paxillin. Integrin acting alone or in complex with growth factors present in the local microenvironment can regulate diverse tumor cell functions, such as migration, invasion, adhesion and proliferation through the activation of various signaling pathways. [created via biorender.com]

The integrins comprise a superfamily of cell adhesion receptors that recognize ECM and cell-surface ligands [15]. There are 18 $\mathrm{\alpha }$-subunits and 8 β-subunits that assemble to create 24 functionally distinct transmembrane heterodimers. Integrins are grouped according to their ligand-binding specificity: collagen-binding integrins (α1β1, α2β1, α10β1, and α11β1), laminin-binding integrins (α3β1, α6β1, α7β1, and α6β4), leukocyte-integrins (α4β1, α9β1, α4β7, αEβ7,αLβ2, αMβ2, αXβ2, and αDβ2) and arginine-glycine-aspartate (RGD)-recognizing integrins (α5β1, α8β1, αVβ1, αVβ3, αVβ5, αVβ6, αVβ8, and αIIbβ3) [15] (Fig. 1a).

These receptor complexes have no enzymatic activity but instead activate bidirectional signaling pathways [13, 15]. The affinity of integrin receptors for ECM components and other ligands is tightly regulated by inside-out signaling [15] (Fig. 1b). Integrin receptors maintain α and β subunit cytoplasmic tail association during their inactive stage, and cytoplasmic signals from associated G-protein coupled receptors lead integrin β subunit phosphorylation within its cytoplasmic domain for receptor activation [15, 16]. The integrin binding of cytoskeletal proteins such as talin, vinculin and ERM (ezrin, radixin, and moesin) acts to regulate actin microfilaments of the cytoskeleton [15, 16]. In contrast, outside-in signaling occurs through the clustering of integrin receptors at the plasma membrane where ECM ligation transduces signals intracellularly [15, 17] (Fig. 1b). Natural extracellular ligands include several components of the ECM such as collagen, laminin, fibronectin and vitronectin [13, 15]. Extracellular ligand binding induces conformational changes in the integrin receptor to allow intracellular tails of the β subunit to engage with intracellular signaling molecules including focal adhesion kinase (FAK), small GTPases Rho and Ras, and adaptors, such as Cas/Crk and paxillin [17]. These activated integrin-ECM interactions lead to the formation of dynamic adhesion structures as small extensions from the plasma membrane called podosomes [15, 16, 18]. After ECM ligation, integrins acting alone or in complex with growth factors present in the local microenvironment can regulate diverse tumor cell functions, such as migration, invasion, adhesion and proliferation through the activation of various signaling pathways [13, 15].

## Implication of integrin function in EOC metastasis

The primary site of origin for HGSOC is the secretory epithelium of the distal fallopian tube from precursor STIC lesions [7, 19]. After *TP53* mutations occur, it is postulated that cells within precursor lesions can be further stimulated by local inflammatory cytokines, growth factors and hormones, such as transforming growth factor-beta (TGF-β) and activin A present in follicular fluid that promote migration of STIC cells to the ovary [20–22]. The movement of STIC lesions to the rich microenvironment provided by the ovary is a critical step in the transition of STIC lesions to HGSOC by attaching, invading and establishing a primary tumor [1, 23–25]. This model has been supported by studies in which ovariectomies performed in mice harboring precursor lesions results in reduced tumor formation and intraperitoneal metastasis, emphasizing the importance of the ovarian microenvironment for complete malignant progression [23, 26].

**Fig. 2:**
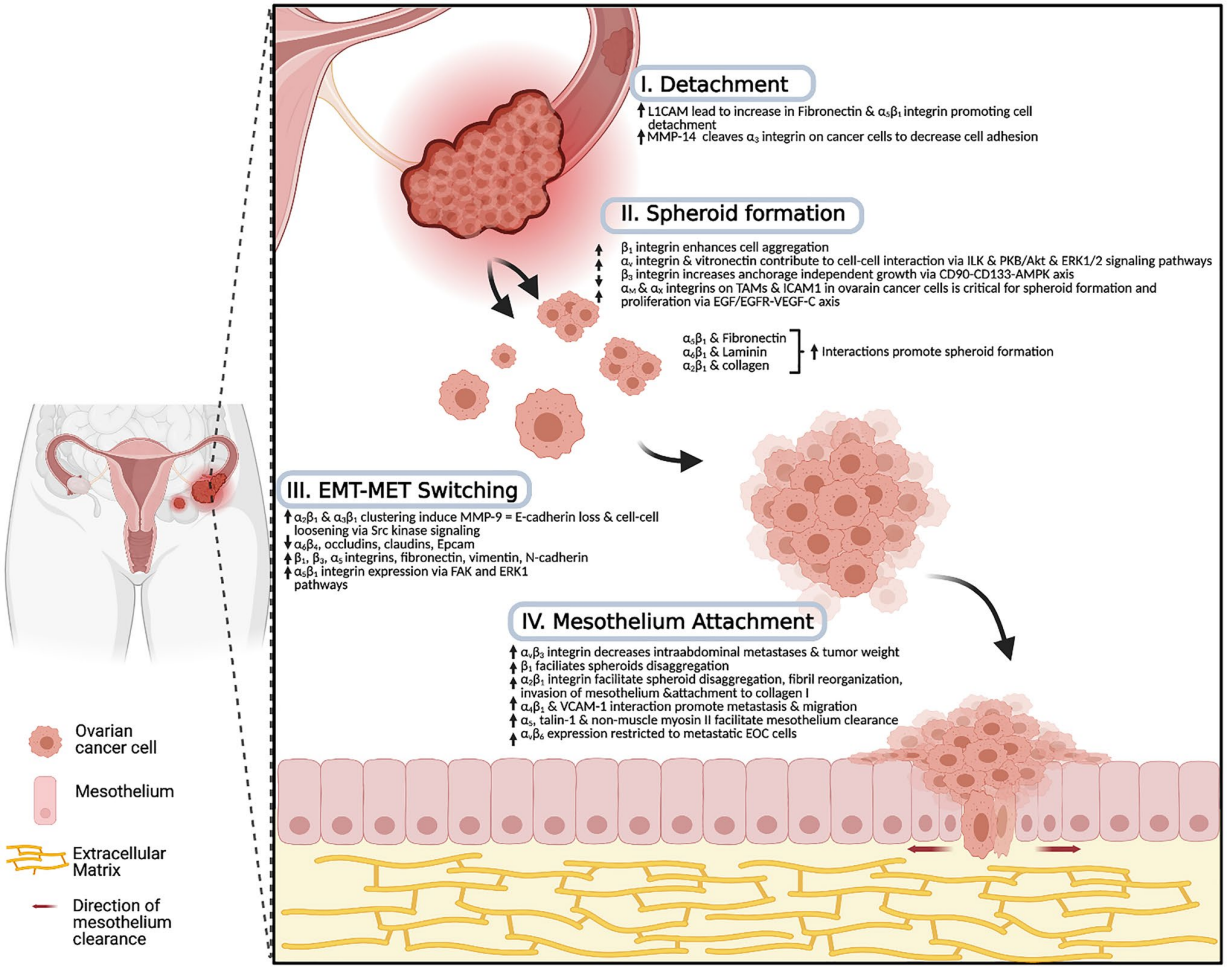
A schematic model of ovarian cancer dissemination and the role of integrins in cancer metastasis. The primary site of origin for HGSOC is the secretory epithelial cells of the distal fallopian tube from precursor STIC lesions and the increase in L1CAM and increased ligation of fibronectin to α5β1-integrin promotes cell detachment. Detached EOC cells survive in hypoxic and anchorage-independent conditions by forming heterogenous multicellular structures known as spheroids to avoid anoikis. Spheroid formation can be enhanced with the interaction of integrins with various ECM proteins. Clustering of integrins such as α2β1 and α3β1 leads to the loosening of intercellular adhesions between cells and contributes to EMT-MET switching. The final step of EOC metastasis occurs when spheroids penetrate mesothelial surfaces, and integrin-mediated degradation of the ECM within the basement membrane underlying the peritoneum leads to secondary metastasis. [created by biorender.com]

Unlike hematogenous routes involving intravasation and extravasation where cancer cells must penetrate multiple barriers, intraperitoneal dissemination is the primary means of EOC metastasis, and is observed albeit less frequently in colorectal, gastric and pancreatic cancers, also [5, 27, 28]. Despite the more passive nature of intraperitoneal dissemination, it leads to rapid disease progression, frequent relapse, complications like bowel obstruction, and overall poor prognosis [5, 6]. During advanced-stage EOC, metastatic cancer cells impair lymphatic drainage and secrete VEGF that enhances vascular permeability and ascites fluid accumulation in the peritoneal cavity [5, 29]. Ascites fluid often contains EOC cells, as well as a highly heterogeneous and variable mix of other cellular and acellular components [5, 9]. Direct spread of EOC tumor cells into the peritoneal cavity is due to enhanced anchorage-independent tumor cell survival that may be supported by altered cell–cell and cell-ECM functions of various integrins (Fig. 2).

### Integrins in EOC spheroids

EOC cells that are bound for metastatic dissemination must first detach from the primary tumor site. Proteolytic activity by membrane type 1 matrix metalloproteinase (MT1-MMP, or MMP14) is required in part for the initial detachment of EOC cells from the primary ovarian tumor by cleaving α3-integrin on cancer cells and contributing to decreased cell adhesion [30]. Detached EOC cells survive in hypoxic and anchorage-independent conditions by forming heterogeneous multicellular structures known as spheroids thereby avoiding anoikis, which is a specific form of apoptosis triggered by the lack of attachment to other cells or ECM [5, 31, 32]. Spheroids further complicate the disease by exhibiting decreased cell proliferation by accumulating in the G0/G1 phase of the cell cycle and becoming resistant to chemotherapeutic agents, such as paclitaxel and cisplatin [33, 34]. It is important to appreciate, however, that not all cells within spheroids may have the same properties. For example, spheroids represent metastatic EOC cells that move in a coordinated fashion, a process called collective migration. This is driven by outer “leader” cells that direct migration and enable the invasion of basement membrane, then “follower cells” that mediate actomyosin contraction allowing for cellular movement [35, 36].

The first step of spheroid formation consists of cell–cell interactions either directly or through ECM bridges. The abundance of integrins available to cells within these multicellular aggregates may provide a major contribution to spheroid formation and pro-survival signaling [37]. Doberstein et al*.* demonstrated that the loss of L1 cell adhesion molecule (L1CAM) reduced the capacity for OVCAR8 cells to form spheroids, ultimately leading to cell death [26]. Alternatively, L1CAM overexpression led to increased spheroid formation in OVCAR8 cells as well as multiple immortalized human fallopian tube epithelial cell lines [26]. L1CAM expression promotes the upregulation of fibronectin and integrin subunits α5 and β1, which together promote cell aggregation into spheroids yet detachment from the primary tumor and tumorigenesis [26]. Fibronectin is abundant within the ascites and plays a critical role in spheroid integrity [38, 39] by interacting with its canonical integrin receptor α5β1 [40, 41]. For example, OVCAR5 spheroids co-express of α5β1 integrin and fibronectin on the their surface [41]. The functional inhibition of β1-integrin using an inhibitory antibody results in the disruption of EOC spheroids, whereas β1-integrin clustering and fibronectin activate α5β1 heterodimer assembly to promote spheroid formation [41].

Spheroid compaction into dense aggregates is critical for tumorigenesis and related to their actomyosin contractile capacity mediated by integrins and cadherins [39, 42]. A positive correlation may exist between compact spheroid formation and tumorigenic capacity, as well as enhanced invasive capacity in EOC [42]. Sodek et al*.* demonstrated that β1-integrin activation using an activating antibody 12G10 and ectopic β1-integrin upregulation enhanced more compact cell aggregation using SKOV3 and OVCAR3 cells, two EOC cell lines that typically form less compact spheroids [42]. Conversely, treating compact HEY cells spheroids with a β1-integrin blocking antibody MAB13 led to spheroid disaggregation [42]. Casey and colleagues also demonstrated that treating OVCAR5 cells with a β1-integrin blocking antibody inhibits spheroid formation, while addition of exogenous fibronectin promoted EOC spheroid formation [41]. Similarly, laminin interactions with α6β1-integrins, and collagen with α2β1-integrins, mediate spheroid formation [41, 43]. In contrast, spheroid formation can be enhanced in the presence of antibodies targeting α2, α4, α6 or αvβ3-integrins, implicating these specific integrins may negatively regulate spheroid formation [41].

Kellouche and colleagues identified αV-integrin and vitronectin colocalization within multicellular aggregates at intercellular sites suggesting a contribution in cell–cell interactions [44]. They demonstrated through the use of anti-vitronectin, anti-αV-integrin, or the cyclic peptide cRGDfV, blocked initial formation of IGROV1 spheroids [44]. The blockade of αV-integrin decreased integrin-linked kinase (ILK) activity resulting in reduced Akt phosphorylation and increased cell cycle inhibitor p27kip1 expression [45]. αV-integrin can directly regulate ILK activity since anti-αV-integrin inhibits ILK activity whereas ectopic ILK overexpression rescues the effect of this blockade [45]. Anchorage-independent growth of IGROV1 spheroid cells leads to a significant decrease in ERK1/2 phosphorylation compared to adherent cells. Inhibition of ERK1/2 activation using MEK1/2 inhibitor U0126 in IGROV1 spheroids decreases cell viability with increased PARP cleavage and caspase-3 activity [46]. A role for integrins in this process was demonstrated by Carduner et al*.* where increased anoikis in IGROV1 spheroids due to αV-integrin silencing is associated with decreased ERK1/2 activation. This suggests that αV-integrin promotes spheroid cell survival by inducing ERK-dependent pathways [46]. This association has also been demonstrated in an anoikis-resistant population of human intestinal carcinoma cells due to αVβ3-integrin expression [47].

Cancer stem-like cells (CSC) may play a role in EOC spheroid formation. CD90, also referred to as Thy-1, is a glycoprotein typically mediating T-cell activation and neurite outgrowth [48, 49]. However, it has been identified as a marker for CSC in gastric, lung, esophageal and liver cancer [50–53], yet its expression is decreased in ovarian cancer tissue and this lower expression of CD90 predicts poor prognosis [51]. Furthermore, CD90 functions as a tumor suppressor gene in ovarian cancer and the expression of CD90 promotes anoikis and inhibits stemness properties including sphere-forming ability [51]. In fact, Chen et al. showed that exogenous CD90 decreases SKOV3 spheroid formation and promotes apoptosis with increased cleaved PARP expression [51]. Ectopic CD90 expression decreases expression of canonical CSC markers CD133 and CD24, and promoted mammalian/mechanistic target of rapamycin (mTOR) phosphorylation and AMP-activated protein kinase (AMPK) [51]. However, β3-integrin silencing reversed the effect by increasing anchorage-independent growth and CD133 marker expression. CD90 is associated with αVβ3-integrin through its regulation of signal transduction in astrocytes and neuronal cells [54]. Taken together, this suggests negative regulatory role of CD90 together with β3-integrin signaling in the context of CSCs and the EOC spheroid phenotype.

Spheroids present in malignant ascites can interact with other cell types to affect their phenotype. For example, an analysis of cell components in spheroids isolated from the ascites of 128 patients with stage III ovarian cancer showed the presence of macrophages in all spheroids [55]. The number of macrophages present with spheroids compared to primary tumors was substantially higher and positively correlated with proliferation in spheroids and negatively with patient prognosis [55]. Robinson-Smith et al*.* demonstrated spheroid implantation in a mouse model of EOC increased due to inflammation, whereas the loss of peritoneal macrophages reduced metastatic potential, supporting the role of tumor-associated macrophages (TAMs) in EOC progression [56]. EOC spheroid cells express intercellular adhesion molecule 1 (ICAM1), a ligand that binds leukocyte-associated integrin subunits αM and αX. Blockade of this interaction between EOC cells and TAMs diminishes spheroid formation in both mouse and human in vitro spheroid co-culture models [55]. TAMs are a source of epidermal growth factor (EGF), and EGF signaling is critical for EOC cell proliferation to increase VEGF-C and enhance integrin-ICAM1 expression, spheroid formation and migration [55].

### Integrins in epithelial-mesenchymal transition and EOC cell migration

Carcinoma cells destined for dissemination oftentimes co-express epithelial and mesenchymal markers, commonly referred to as epithelial-mesenchymal transition (EMT), allowing for a cadherin switch [36, 57]. EMT allows ovarian cancer cells to loosen intercellular adhesions between cells contributing to the transition of cells from a primary tumor to shed as single cells or spheroids into the ascites [5, 39]. During this EMT process, EOC cells gain enhanced invasive properties, survive in hypoxic conditions, and spread through the abdominal cavity by the peritoneal fluid flow [57, 58]. The decrease in cell–cell adhesion and detachment of EOC cells from the primary tumor into the peritoneal cavity is mediated through the integrin-mediated upregulation of matrix metalloproteinases (MMPs) and activation of EMT [36]. Clustering of collagen-binding integrins α2β1 and α3β1 on EOC cells leads to the induction of MMP9, which is capable of E-cadherin ectodomain cleavage and cell–cell adhesion loosening in an Src kinase-dependent manner [59]. E-cadherin loss leads to transcriptional upregulation of fibronectin receptor α5β1 integrin, which is essential when spheroids initiate adhesion at a secondary site [60]. Decreased E-cadherin is also accompanied by reductions in occludins, claudins, epithelial cell adhesion molecule (EpCAM), α6β4-integrin and cytokines, all of which act to stabilize tight cell–cell contacts via desmosomes [22]. In a reciprocal fashion, there are increases in vimentin, fibronectin, N-cadherin, β1- and β3-integrins and matrix metalloproteinases [22]. Forced downregulation of E-cadherin in EOC cells increases α5-integrin expression through FAK and ERK1 activities leading to enhanced cell adhesion to fibronectin [61]. As expected, these EMT-like changes due to E-cadherin loss promote EOC cell invasive properties required for metastasis [58].

TGF-β signaling is widely-recognized as one of the most important pathways required to promote EMT in human cancers. We demonstrated that TGF-β activity is induced during ascites-derived EOC spheroid formation and controls increased mesenchymal marker transcript expression, whereas its inhibition dramatically reduces EMT properties and cell–cell cohesion within spheroids [62]. In breast cancer, Bianchi et al*.* have shown that β-integrin subunits associated with αV-integrin are upregulated during TGF-β-induced EMT, and the specific downregulation of β5-integrin blocks this effect [63]. Similarly, αVβ8-integrin mediates latent TGF-β activation and resultant EMT in various cancers contributing to cell migration and growth [64].

The ascites microenvironment within which EOC cells and spheroids reside may play a critical role in promoting a partial shift towards their mesenchymal phenotype [46]. When cultured EOC cells are exposed to ascites, αV-integrin localization moves from focal contact structures to intracellular perinuclear vesicles in IGROV1 cells [46]. Furthermore, the αV-integrin cyclic antagonist cRGDfc peptide inhibited multicellular aggregate formation by 40% compared to a non-targeting control peptide [46]. Taken together, these studies suggest that αV-integrin and TGF-β work in concert to control EMT, cell adhesion and migration, within the context of EOC spheroids; however, a broader role of αv-integrin complexes in EOC pathogenesis remains unclear and further investigation is required.

### Integrin-mediated mesothelial attachment and migration

When establishing secondary tumors, spheroids attach to the mesothelium lining through the interactions between spheroids and surface receptors on the mesothelial layer [22, 65]. At this point, spheroid cells induce expression of several integrins that prime the spheroids for attachment to the mesothelium and underlying ECM proteins [22, 41, 66]. For example, interaction between spheroid cells expressing α5β1-integrin receptor and the mesothelium containing fibronectin matrix is essential for spheroid adhesion [41]. Although Casey et al*.* showed that neutralizing antibodies against α5β1- and αVβ3-integrins did not affect the binding of EOC cells to the mesothelium, additional studies by this group and others have shown a partial block in adhesion when inhibitory β1-integrin antibody was administered [41, 67, 68]. Similarly, inhibition of α3-, α6- and β1-integrin subunits decrease invasiveness and collagen-binding of spheroids [36]. As the major receptors for ECM proteins, integrins are critical regulators of EOC cell adhesion and invasion at a secondary site.

The final step of EOC metastasis occurs when spheroids penetrate mesothelial surfaces and degrade the ECM within the basement membrane underlying the peritoneum, omentum and abdominal organs [69]. Kaur et al*.* demonstrated that β3-integrin expression correlates with increased EOC cell adhesion in vitro and adhesion to mesothelium and mouse omentum in vivo [70]*.* However, they also showed that αVβ3-integrin overexpressing cells inhibited invasion using Matrigel, and β3-integrin blockade resulted in enhanced invasion in CAOV3 and MONTY1 cells [70]. These latter results were recapitulated in vivo where αVβ3-integrin overexpressing cells displayed a 35% decrease in intra-abdominal metastases and 53% decrease in tumor weight compared to controls [70]. These results highlight the potential mechanistic differences involving integrins between EOC cell adhesion, invasion, and successful secondary tumor establishment.

Integrins function in both cell–cell adhesion and binding to basement membrane and ligand components [69]. α2- and β1-integrin subunits contribute to EOC cell adhesion via collagen I facilitating peritoneal attachment and invasion into the mesothelial monolayers [71]. Several studies have shown that inhibition of collagen-associated α2β1-integrins leads to attenuated spheroid disaggregation on artificial ECM since primary EOC cells adhere to type I collagens preferentially [59, 67, 72]. Furthermore, Davidson et al*.* showed high expression of αV- and β1-integrin subunits in malignant cells from peritoneal and pleural effusions collected from late-stage EOC patients suggesting a potential role in metastasis [73]. The interaction between vascular cell adhesion molecule 1 (VCAM-1) present on the mesothelium and α4β1-integrins on EOC cells promotes metastasis and cell migration in xenograft models [69]. Indeed, this study also demonstrated that the use of function-blocking antibodies against either VCAM-1 or α4β1-integrins show promise in decreasing EOC metastasis [74].

Recent studies suggest that EOC cells may not adhere directly to mesothelial cells, but rather to underlying connective tissue; this is achieved by disrupting cell–cell junctions, a process called mesothelial cell clearance [75]. Using highly innovative live-cell imaging techniques, Iwanicki et al. demonstrated that EOC spheroids use integrin- and talin-dependent activation of myosin traction force to promote mesothelial cell clearance [75]. In this experimental model, mesothelial cell monolayers were plated on fibronectin-coated polyacrylamide gels to mimic physiologically-relevant stiffness of connective tissue. They showed that OVCA433 spheroids induced mesothelial clearance by the above mechanisms [75]. As spheroids promote mesothelial clearance, fibronectin fibrils organized on top of mesothelial cells are redistributed away from between the mesothelium and attaching spheroids [75]. Blocking of α5-integrin, talin 1 and non-muscle myosin II abrogated mesothelial displacement, while ectopic expression of α5-integrin increased myosin-mediated cell spreading, stress fibers, and other cortical actin contractile structures [75]. Collagen I-associated α2-integrin subunit induced fibril reorganization and transmitted traction forces to ECM, but spheroids expressing high levels of α2-integrin rather than α5-integrin were unable to clear the mesothelium [75]. Interestingly, blockade of another fibronectin receptor αVβ3-integrins did not affect mesothelial clearance, suggesting these specific receptors do not have a myosin-contractility roles in EOC metastasis [75]. In a different study, Kokenyesi et al*.* reported SKOV3 and OVCAR3 cells were unable to invade a collagen I matrix due to their inability to disrupt intercellular interactions, highlighting the importance of integrin-mediated actomyosin contraction to overcome cell–cell attachments [76].

A critical component of tumor invasion is enzymatic degradation of the ECM, which permits cancer cells to penetrate the basement membrane, gain access to the vasculature and successful formation of secondary tumor growth [40]. Spheroid cell migration using OVCAR5 cells on laminin- and collagen IV-coated surfaces showed a modest twofold change in cell migration over 24 h, whereas spheroids on fibronectin and collagen I completely disaggregated to form a monolayer with a ninefold change in surface area [40, 66]. Addition of an inhibitory antibody against β1-integrin completely eliminated spheroid cell migration on laminin, fibronectin and type-IV collagen, and a 50% reduction on type-I collagen [40, 66]. However, these results suggest that β1-integrin blockade did not prevent initial spheroid attachment, but rather significantly impacted ECM degradation and spheroid disaggregation of invading foci [40]. Overall, β1-integrins partially mediate adhesion of EOC spheroid cells to ECM, but it likely plays a more significant role in ECM degradation to promote spheroid disaggregation [40, 66].

MMPs are zinc-dependent proteinases that degrade various ECM components, such as collagens, proteoglycans, gelatins, vitronectin, and fibronectin [77]. EOC cells that express higher levels of MMP2 and MMP9 possess increased invasive and metastatic potential [72, 78]. Studies by Kenny et al*.* demonstrated that contact of EOC cells with mesothelium induces MMP2 expression at the transcriptional and translational levels [79]. Activated MMP2 cleaves various ECM components, including vitronectin and fibronectin, into smaller fragments to improve EOC cell adhesion to αVβ3- and α5β1-integrin receptors [79]. αVβ3- and α5β1-integrin blocking antibodies inhibited cell adhesion, however, this effect was abolished when EOC cells were preincubated with MMP2 antibody [79].

When cells migrate away from the core of an attached spheroid, cell–cell contacts are reduced while adhesion and spreading on ECM occurs. Compared to monolayer culture, MMP2 and MMP9 activities are increased in serum-free medium collected from spheroid culture [72]. Interestingly, Shield et al*.* demonstrated OVHS1 and HEY spheroids have reduced disaggregation in the presence of α2β1-integrin with a coordinated reduction in active MMP2 levels [72]. The reciprocal expression of increased α2-integrin and decreased α6-integrin subunits in OVHS1 and HEY spheroids were also observed.

Furthermore, αVβ6-integrin in EOC is correlated with increased expression and secretion of high molecular weight-urokinase-type plasminogen activator (uPA), pro-MMP2 and pro-MMP9, in tumor-conditioned media [78]. Interestingly, αVβ6-integrin expression is restricted to metastatic EOC cells with little to no expression in benign and normal ovarian epithelial cells [78]. Results from this same study demonstrate that αVβ6-integrin-expressing EOC cell lines have an enhanced capacity to degrade the basement membrane in a plasminogen-dependent manner since this effect was completely abolished by inhibition of uPA, MMP9, or αVβ6-integrins [78].

## Integrins as therapeutic targets for EOC

Novel therapies that focus on malignant cells and their interactions with the tumor microenvironment in EOC have gained substantial interest due to the complexity of this disease. As described in detail above, integrins are key regulators at various steps in the unique metastatic cascade of EOC, particularly during the process of spheroid formation during transit, and for peritoneal invasion where integrin-ECM interactions are essential during spheroid adhesion and invasion [36].

Preclinical and clinical studies of integrin antagonists show promising results to effectively block tumor progression [80]. Integrin inhibitors represent a feasible therapeutic strategy since the majority of Phase I clinical trials demonstrate that these agents are well-tolerated by patients in conjunction with cytotoxic chemotherapy or radiotherapy [13, 36]. The chimeric monoclonal antibody Volociximab targets α5β1-integrin and has been successful in inhibiting angiogenesis and impairment of tumor growth [77]. Preclinical data shows that intraperitoneal treatment of SKOV3ip1 xenografted mice with Volociximab reduced tumor burden and ascites accumulation by 83% and 97%, respectively [81]. Encouragingly, clinical trials showed that EOC patients with platinum-resistant disease treated with a weekly administration of Volociximab was well-tolerated [82]. ATN-161 is a non-RGD based synthetic pentapeptide derived from fibronectin that binds to and blocks both α5β1- and αVβ3-integrins [83]. This agent has shown promise using mouse xenograft models of breast cancer metastasis and it was safe in patients with stable disease, but has not been tested in EOC patients yet [84, 85]. Etaracizumab is an anti-human monoclonal antibody against αVβ3-integrin developed after encouraging preclinical results showing decreased tumor burden in SKOV3ip1 and HEYA8 xenograft mouse models [86]. However, clinical trials showed minimal effectiveness as a therapeutic treatment for metastatic disease [83]. Another humanized antibody Intetumumab targeting αVβ3- and αVβ5-integrins showed effective inhibition of cancer cell adhesion and migration of six different uterine serous papillary carcinoma cell lines in vitro [87]. Phase I clinical trials show that it is safe, it localized to tumors, and it exhibited some signs of anti-tumor activity, but these early findings require additional trials [88]. Although promising results have been seen with anti-integrin αVβ3 antibodies, results from Kaur et al*.* suggest that of overexpression of this integrin complex correlates with a favorable patient outcome; thus, further clinical investigations are required [70].

Several different approaches for targeting integrins may offer therapeutic potential in the future, however no single integrin receptor complex inhibition strategy has shown sufficient clinical trials data to progress for further investigation yet [36]. One major hurdle impeding success may be the complexity and dynamics of integrin functions implicated in EOC tumor growth and metastasis (Fig. 2). For instance, the crucial interaction of EOC cells for adhesion via fibronectin is not limited to α5β1-integrin, as αVβ3- or α3β1-integrins can compensate for its loss-of-function [36]. Adding to this challenge will be the broad intratumor and interpatient heterogeneity in this disease. Insight into the cellular mechanisms involved in cancer cell survival and progression through in vitro and in vivo models are critical to the development of cancer therapy. As such, the complex intratumoral and interpatient heterogeneity in EOC can be addressed experimentally through the use of patient-derived organoid (PDO) models that retain many features of primary tumors [89]. This relatively new three-dimensional tumor model will be important to further address integrin-mediated function on disease pathogenesis, particularly when compared with the established evidence using spheroids in suspension.

In conclusion, integrin expression and function are clearly implicated at numerous steps of EOC metastasis, most notably as spheroid cells in the peritoneal cavity and their subsequent attachment and invasion at secondary sites [90]. As mediators of EOC metastasis to the mesothelium, the use of specific integrin inhibitors alone or in combination could prove effective in preventing sustained interaction between EOC cells and mesothelium. Taken together, we foresee that a combined approach of targeting multiple integrin-associated pathways may be worthy of future exploration in both experimental and clinical applications.
